# Herding Ecologies and Ongoing Plant Domestication Processes in the Americas

**DOI:** 10.3389/fpls.2018.00649

**Published:** 2018-05-17

**Authors:** Paulina R. Lezama-Núñez, Dídac Santos-Fita, José R. Vallejo

**Affiliations:** ^1^Red Conbiand Mexico, Mexico City, Mexico; ^2^Asociación Etnobiológica Mexicana A.C., San Cristóbal de las Casas, Mexico; ^3^Área de Didáctica de las Ciencias Experimentales, Equipo de Historia de la Ciencia y Antropología de la Salud, Facultad de Educación, Universidad de Extremadura, Badajoz, Spain

**Keywords:** management, pastoralism, niche, animal agency, maize and quinoa agriculture, rituality, American continent

## Abstract

Understanding both domestication processes and agricultural practices is an interdisciplinary endeavor. Ethnographic research is potentially helpful for reconstructing past events. Such knowledge is also crucial for documenting the links between biological and cultural diversity, as well as for future purposes such as innovation in food production and sustainability. Here, we review six ethnographic case studies in different pastoral socioecological systems of the American continent. The livestock species involved include the native South American camelids and Arctic reindeer, as well as some Old World species (mainly goats, sheep, and cattle). Starting with the Columbian exchange (15th-16th centuries) and continuing up to the present, Old World herbivores launched novel uses of the local flora which resulted in entirely new livelihoods and cultures, i.e., pastoralism with its variants. Three of these case studies approach specifically how herding ecologies (human–animal–plant relationships) stirred specific management practices (human–plant relationships) that in some instances have moved toward conscious human selection of plant phenotypes. The other examples correspond to three potential instances of similar ongoing processes that we propose on the basis of ethnobotanical and ethnozoological data that were produced separately by other authors. Based on the studies we have reviewed, along with additional information from other parts of the world, we are able to conclude that: (a) New World pastoralist societies are/have been continuously adding species to the humanity’s portfolio of useful plants; (b) animals have been aiding in this processes in different ways; and, (c) how human–animal–plant relationships unfold in the present could have been similar in the past, thus analogies may be proposed for explaining prehistoric multispecies interactions and their outcomes. With our review, we intend to bring more attention to contemporary pastoralists as plant managers, animals as agents in human-plant interactions, and domestication as a behavioral complex and multispecies process that is as important in the present or future as it was in the past. Our understanding of food production practices is not only fundamental for improving our current frameworks of governance, conservation, and restoration of useful species populations, but also of biocultural diversity altogether.

## Introduction

Domestication studies cover instances of human niche construction at a wide range of space and time, as well as different scales of analyses and analytical approaches. The vast majority of this research focuses on initial domestication and on overreaching explanations of the very inception of such human evolutionary trajectory. As discussed by different authors (Larson et al., 2014; Vigne, 2015; Bar-Yosef, 2016) some of the questions regarding the so-called Neolithic Revolution (around 10,000 years ago) are easier to answer than others. Overall, one can find more agreement on ‘where’ and ‘when’ matters. At the same time, however, the centers of origin and movement of domesticates are continuously refined and reworked (Larson and Burger, 2013; Fuller et al., 2014; Zeder, 2015). In contrast, consensus is more difficult to reach in the answers to the ‘how’ and ‘why’ questions of agricultural origins and species domestication. While the interaction of various factors and their changes through time and space are acknowledged by most scholars, disagreements appear in the form of, for example, ‘pull’ *vs.* ‘push models’ (Hayden, 2009; Bowles and Choi, 2013; Gremillion et al., 2014; Willerslev et al., 2015), ‘model driven’ *vs.* ‘particularism’ or non-model explanations (Zeder and Smith, 2009; Vigne, 2011; Gremillion et al., 2014), or the ‘centric model’ *vs.* ‘non-centric model’ for the characterization of domestication processes (Fuller, 2010; Langlie et al., 2014; Abbo and Gopher, 2017).

An additional body of literature is concerned with domestication processes occurring at recent ecological scales rather than in the distant past. Such studies have been useful in different ways to our understanding of agricultural practices. For one, they have underlined that human creativity is great in generating and adopting diverse subsistence practices in similar physical environments throughout the globe and history. For another, scholars using ethnohistoric and ethnographic sources have described and categorized the extent of peoples’ practices driving their interactions with other species and the environment (Casas et al., 2007; Hildebrand, 2009; Lira et al., 2016). This work has served to inform the reconstruction of economic and other activities of our forebears. Although one has to be aware that ethnographic analogies can only be a guide, they rightly inform archeological approaches as well (Hildebrand, 2009).

On the specific question of animal and plant domestication, virtually all research, of both ancient and recent processes, covers either plant or animal species separately (for global reviews see Zeder, 2012; Larson et al., 2014; Vigne, 2015; Bar-Yosef, 2016; for the Americas see Casas et al., 2007; Pearsall, 2008; Stahl, 2008; Clement, 2014). Ironically, domestication research of either animal or plant populations is carried out against a background of full recognition that human activities affect local ecologies as a whole, and that indigenous peoples modify their environments not upon a perception of discrete and unrelated biological entities, but based on an inseparable complex of knowledge, practice and beliefs (Berkes, 2008). Although domestication literature often mentions that, in those places where both animals and plants were domesticated the respective processes influenced each other, giving birth to the so-called ‘agricultural packages’ of Asia, Northern Africa, or the Andes, little or no detail is given of how this could have happened. For instance, it is expressed that sometimes either plant or animal domestic species appeared earlier, later, or simultaneously in the same places (Zeder, 2011; Larson et al., 2014; Vigne, 2015; Bar-Yosef, 2016).

**FIGURE 1:**
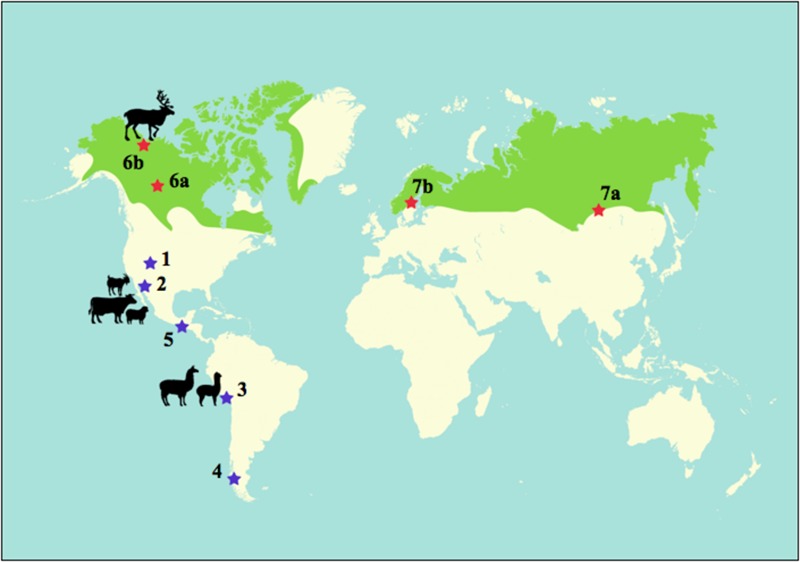
Locations of herding ecologies that have lead or can potentially be leading to landscape and plant domestication. The blue stars indicate mixed flocks of sheep/goats/cattle (1: Navajo, 2: Rarámuri, 4: Mapuche, 5: Tzotzil) or sheep/goats/cattle/camelids (3: Aymara). The green area corresponds to the distribution area of reindeer or caribou (*Rangifer tarandus*); red stars indicate reindeer herding locations (American Arctic with 6a: Shúhtagot’ine, 6b: Gwich’in; and Old World Arctic with 7a: Evenki, 7b: Sami). All the stars also indicate the nine geographic sites of all ethnobotanical and ethnozoological data as also shown in **Tables 1, 2**.

In this paper, we present some exemptions where both realms –plant and animal domestication– are coupled (**Tables 1, 2** and **Figure 1**). Our literature review is concerned with the understanding of how human-animal relationships –in pastoral contexts– can lead to domestication not only at a landscape but also at a species one. On the one hand, these case studies unfold domestication processes at ecological, observable scales; and on the other, they allow a better envision of human niche construction than when only approached at the human–plant or human–animal interface. As mentioned above, we also believe that these examples have the potential to guide hypotheses of similar instances (both past and present) of domestication wherein people have presently or historically depended on herd animals. We have structured the text as follows. First, we define the concepts of landscape and species domestication and briefly review the relevant literature of early herding in the Old World. Second, we provide a short picture of the impacts that alien livestock species (mainly goats and sheep) have had on the biocultural diversity of the New World, followed by the description of three case studies (1: Navajo-United States, 2: Rarámuri-Northern Mexico, and 3: Aymara-Andes) that specifically approach human–animal–plant mutualisms in pastoral contexts (**Table 1**). Third, we gather further ethnobotanical and ethnozoological data for constructing an additional set of case studies (4: Mapuche-Patagonia, 5: Tzotzil-Southern Mexico, and 6: American Arctic) that can potentially render additional evidence of similar mutualisms (**Table 2**). As it can be seen in **Table 2** and **Figure 1**, for the American Arctic case, data from Old World Arctic locations was included. Finally, we discuss the information provided in terms of dominant and alternative narratives of domestication processes, biocultural diversity, and issues of innovation in food production and sustainability.

**Table 1:** Case studies on herding ecologies which have led to landscape and plant domestication.

Culture/Geographic location	Livestock species^a^	Plant species^a^	Reference
Navajo or Diné/Southwestern United States	Sheep	*Cleome lutea, Chenopodium album, Descurainia pinnata, Sisymbrium altissimum, Polygonum aviculare, Amaranthus graecizans, Salsola iberica, Halogeton glomeratus, Malva neglecta, Gilia aggregate, Erodium cicutarium, Nicotiana attenuata*	Kuznar, 2001
Rarámuri or Tarahumara/Sierra Tarahumara, Northern Mexico	Goats, sheep cattle	*Brassica campestris*	Bye, 1979
Aymara/Asana Valley, Southern Peru	Goats, sheep, cattle, camelids	*Chenopodium* spp., *Dunalia brachycantha, Mutisia acuminata, Cantua candelilla*	Kuznar, 1993

*^*a*^The mutualisms among humans (culture), herbivores (livestock species) and plants are enlisted; only the most common species as reported by the authors are provided.*

**Table 2:** Case studies on herding ecologies which can potentially be leading to landscape and plant domestication.

Culture/Geographic location	Livestock species^b^	Plant species^b^	Reference^a^
Mapuche/Southern Argentina	Goats	?^c^	Ladio and Lozada, 2004, 2009; Lanari et al., 2005; Castillo and Ladio, 2017
Tzotzil/Los Altos de Chiapas, Southern Mexico	Sheep	?^c^	Geerlings and Perezgrovas, 2014; Gómez et al., 2014; Perezgrovas et al., 2014
Shúhtagot’ine/Northwest Canada [American Arctic]	Reindeer	?^c^	Andrews et al., 2012
Gwich’in/Yukon (Canada) and Alaska (United States) [American Arctic]	Reindeer	?^c^	Anderson et al., 2017
Evenki/Baikal-Patom plateau, Siberia [Old World Arctic]	Reindeer	Chenopodiaceae, Poaceae, *Polygonom aviculare*	Anderson et al., 2014
Sami/Fennoscandia [Old World Arctic]	Reindeer	*Angelica archangelica*	Rautio et al., 2016

*^*a*^References used for constructing suggested additional case studies consisting of well-studied long-term pastoralism (including both ethnobotanical and ethnozoological studies). ^*b*^The mutualisms among humans (culture), herbivores (livestock species) and plants are enlisted; only the most common species as reported by the authors are provided. ^*c*^The authors provide only either ethnobotanical or ethnozoological data.*

## Domestication and the Pastoral Niche

Conventionally, landscape domestication is defined as a cultural process whereby humans modify their landscapes, affecting the demographics of plant and animal populations, and consequently, local ecologies thus making them more productive and hospitable (Clement, 2014). Species domestication, on the other hand, is defined as a coevolutionary process in which people select particular phenotypes of individual organisms changing both, the phenotypes and genotypes of plant or animal populations, making them more useful and better adapted to human management (Clement, 2014). Other definitions of species domestication acknowledge a more balanced human-domesticate role in the process, as well as the mutual benefits obtained by the partners in the domesticatory relationship; however, they still require some kind of quantification of the changes occurred in the plant or animal domesticates (Zeder, 2015). Here, we adhere to a broader conception of domestication as a behavioral complex of interest in itself, and not only for what it leads to; that is, the expected appearance of the so-called ‘domestication syndromes’ in animal or plant populations. As Terrell et al. (2003, pp. 325–326) explain, “domestication should be measured more by its conduct than by its consequences,” and “any species or place may be called domesticated whenever another species knows how to harvest it.” Under this vision,

“species (and places) do not have to be morphologically or genetically altered or distorted in some clearly discernible way before they may be called domesticated […], concluding that only plants, animals, and places exhibiting plainly detectable signs of use may be labeled ‘domesticated’ risks greatly underestimating the generality and force of domestication in the world around us” (*Op. cit.*).

Importantly, such inclusive definition highlights the facts that changes in animal and plant populations are not only possible under intentional human management, but can also be a consequence of unintentional actions. In addition, it covers those ‘wild’ species that are not staples, but which are highly important in times of failed harvests (e.g., ‘wild greens’), or otherwise essential in local diets (e.g., garnishes, spices), as well as other species of diverse cultural uses (medicinal, religious, etc.).

Therefore, we use the terms ‘domestic’ and ‘wild,’ or ‘foragers’ and ‘farmers,’ as handy ‘tools for thought’ and not as real, pristine categories of neither plants/animals nor human beings. As other authors have discussed, “focusing on precise demarcation of such thresholds […] creates the erroneous impression of dichotomous states […] and distracts attention from the often opaque, but far more interesting, middle-ground areas that lie between them” (Zeder, 2015, p. 3193) (see also Terrell et al., 2003; and discussion in Bellwood et al., 2007, particularly Gamble’s, Pluciennik’s, and Terrell’s viewpoints). Certainly, it is in these ‘opaque’ areas where the early farmers, together with their first ‘agricultural packages,’ and populations at early stages of domestication are located. In addition, the ‘pastoral niche’ that emerged with the concomitant domestication of those plant and animal species is useful for envisioning the multispecific nature of human niche construction. It is toward such multispecies settings where we now turn our attention, and more specifically, we focus on how human-animal relationships in herding ecologies guide both landscape and plant domestication.

### The Pastoral Niche

Only recently, McClure (2015) integrated data of modern breeds (mainly sheep, goats, and cattle) and current pastoral contexts to describe how animal herding creates a ‘pastoral effect’ in human niches. She proposes that similar effects could have been present when/where herding ecologies were first undertaken by people. The pastoral effect in human niche construction is based on: (1) the biological characteristics of the herd animals, and (2) the cultural traits of livestock management. The main processes involved in the human-animal co-construction of the pastoral niche are perturbation (i.e., the modifications that organisms cause on the environment) and relocation (i.e., the movement of an organism to a new place thus being exposed to a new selective environment). The reader is referred to her work to learn the details of her proposal, but here we extract the basic mechanisms that are complementary to our next section (“Animal agency in landscape and plant domestication”).

Both humans and animals begin perturbation activities when foraging in a new area; they first modify plant communities by selective foraging as well as selective reseeding through defecation, but they also affect local flora by trampling and causing additional biophysical changes in the soils. In addition, humans clear vegetation for different purposes and manage plant populations in diverse ways. Then, depending on the species composition of the herds, their size, and how people manage the different plant species surrounding their settlements, differences in germination of the plant species occur. Eventually, different strategies evolve from the dynamics among human, animal, and plant communities, and other socioecological factors allowing for vegetation to regenerate. For example, mobility may emerge in the form of transhumance or nomadism, while sedentary agropastoralism is yet another possibility. In the former cases, the human–animal impact on plant communities over vast landscapes would be different and of different intensity to that of the latter, where the penning and foddering of animals involve extra modifications of plant communities by people. Since fodders can be gathered from the wild but also intentionally grown (e.g., crop by-products, surplus), a direct link between management of plant and animal domesticates can be seen; besides, confinement in corrals facilitates the accumulation of dung that can be used as fuel and fertilizer, which further assists plant cultivation (Bye, 1979; Kuznar, 1993, 2001; McClure, 2015).

As described earlier, not many empirical studies have aimed at determining how the processes by which the domestication of each of the members of the first ‘agricultural packages’ may have influenced one another. There are only a few exceptions to this pattern, including a handful approaching the question in the ancient past (all in the Old World) (reviewed by Langlie et al., 2014) and others following the Columbian exchange (all in the New World). Within the first group, for example, herbaceous seeds assemblages of animal dung remains, alongside other lines of evidence, have helped to reconstruct local subsistence economies where full season pastoralism but no seasonal dry-farming were coupled (at Atar Haroa, Israel) (Shahack-Gross and Finkelstein, 2008; Shahack-Gross, 2011, in Langlie et al., 2014). In other case, stable isotopes of goat bones, on their part, have showed that one of the first methods of animal husbandry consisted of provisioning them with fodder (in the Near East) (Makarewicz and Tuross, 2012, in Langlie et al., 2014). Stable isotopes evidence has also indicated that a significant part of the early domestic pigs’ diet consisted of millet (in China), which led to propose that cereal and animal domestication reinforced each other (Chen et al., 2014, in Langlie et al., 2014). All these data conform to McClure’s (2015) reconstruction of the first European pastoral niches. Interestingly, Svizzero (2016) has posed the hypothesis that animal domestication, specifically of sheep and goats, was initiated not only via selective hunting (as usually claimed), but also by baiting; and more specifically, through the cultivation of food plots for attracting wild game, either to kill them or to capture them alive. Svizzero states,

“baiting is hardly considered in the academic literature about the Neolithic revolution. We believe that it is so because the transition to farming is most of the time considered from farmers’ point of view. [It] must be considered from the point of view of those who managed these wild resources, i.e., from hunters’ point of view” (Svizzero, 2016, p. 55).

To our knowledge, his proposal is the explanation that provides the most detail as to how plant management could have led to animal domestication in this part of the world.

In the second group of related works we find the mirror image of Svizzero’s theory: that camelid management could have led to the domestication of an indigenous Andean crop, *quinoa*, approximately 3,000 years ago. Such hypothesis, however, is based on recent empirical evidence (Kuznar, 1993; Fritz et al., 2017). Parallel evidence suggesting that animal husbandry promotes plant domestication processes has also been found in the North American, Southwest (Kuznar, 2001) and Mesoamerican pastoral contexts (Bye, 1979). In the latter example, such ongoing processes involve conscious human selection of plant phenotypes. In all three cases, the herding ecologies include only or mostly Old World herbivores (mainly goats and sheep) (see **Table 1**).

### Animal Agency in Landscape and Plant Domestication

We believe that herding ecologies give birth to certain human-plant mutualisms by means of two related mechanisms. The first is indirect or secondary, by co-creating the anthropogenic environments, i.e., the pastoral niche, where certain plant species thrive at the expense of others while also dispersing seeds in/out settlements (McClure, 2015). The second is direct or primary, by bringing human attention to certain flora, i.e., pastoralists establish intimate relationships with their herds that allow them to first recognize and then experiment with the plants that their animals themselves are consuming. These mechanisms would be complementary to the views of plant domestication as an unconscious evolutionary process (non-centric) (Fuller, 2010), or a knowledge-based human initiative (centric) process (Abbo and Gopher, 2017; for a review, see Langlie et al., 2014).

The indirect animal impact on human–plant relationships is observable at both the landscape and species levels. On the one hand, pastoral anthropogenic environments are created by the unintended biophysical changes that both human and animal disturbances create in conjunction. Landscape domestication, i.e., the changes of species composition in plant communities, takes place as a consequence of the jointly, always concurrent and inseparable activity of pastoralists and their herds. Importantly, there is a wealth of academic literature focusing on the animals’ agency when choosing their food (e.g., flexibility, preferences, social learning) and how this affects plant communities (Villalba and Provenza, 2009; McClure, 2015). Thus, livestock species are themselves domesticators of their shared habitats with humans. On the other, plant domestication occurs when people start managing any species of their pastoral niches. This happens after landscape domestication, when humans begin closer and more frequent encounters with the new plant communities in and around their pastoral settlements. In at least one of the cases reported here (2: Rarámuri), agropastoralists have embarked on the conscious selection of certain phenotypes of a particular species (mustard, *Brassica campestris*). In other two cases (1: Navajo and 3: Aymara) a range of cultural uses have been reported for some of the dominant plants found near the villages (food, fodder, ritual), or can be inferred from ethnobotanical reports (4: Mapuche, 5: Tzotzil, and 6: American Arctic). In addition, practicing transhumance means being exposed to higher species richness and a larger number of plants *vs.* those who do not travel (4: Mapuche). The legacies of such human–animal–plant interactions have been detected even after only a few centuries of pastoralism in the New World (next section; and **Tables 1, 2**).

The direct animal impact on human–plant relationships is detectable at the species level as well. Here, however, human and animal intentions become intermingled in varying and fascinating ways: not only do people notice those species most common in their environment but they also remain attentive to their animals’ preferences at all times and continuously learn from them. Pastoralists and other indigenous peoples around the world gain understanding of the myriad of ecological interactions in their local environments, like plant biology and ecology, by watching other organisms, regardless of the wild/domestic status of the species they coexist with (Nabhan, 2000; Huffman, 2003). From ethnographic evidence one can attest the constant and careful monitoring that people practice over their herds (Perezgrovas, 2004; Wyndham, 2009; Perezgrovas et al., 2014; Molnar, 2017). Moreover, pastoralists are well aware of their animals’ abilities for self-medication (Huffman, 2003; Villalba et al., 2005). From this, we hypothesize that at least some of the species that people have learned to use (i.e., domesticate) were at first tried because they saw livestock species consuming them. Simultaneously, observing herd animals and their ecological interactions may have aided humans in learning how to cultivate certain plants. In this way, plant discovery and plant management can be seen as an interspecific learning process, and not only as a pure human achievement. Indeed, from the herders’ perspective, they are indebted to their animals for their knowledge of landscapes and plants, as can be seen in their explanations such as that they see the grass through the mouth of their animals (Wyndham, 2009; Anderson et al., 2014; Molnar, 2017). Although in the studies we will review next, no instance of such direct animal impact on human–plant relationships was reported, here we suggest that given the circumstantial evidence found in these and other countless ethnobotanical/ethnoveterinary surveys, the potential for searching those kinds of motivations is high: significantly, the large overlap of human/animal useful flora is extensive across the globe, with those species of weedy tendencies being particularly salient (for the Old World, see Zohary et al., 2012; for the New World, see Lira et al., 2016). We believe that it is of high importance that in future enquiries particular attention and further thought be given to these kinds of pastoralists’ interpretations. This would very likely provide a deeper understanding of how people established and continue to establish relationships with plants.

We now present six case studies in the Americas that show the indirect animal impact on human–plant relationships (**Tables 1, 2**). First, we review instances where Old World livestock have contributed to both cultural endurance and plant diversity enhancement through domestication. Then, we do the same for Andean and Arctic landscapes where camelids and reindeer, respectively, have supported some Native American cultures for millennia. It is important to note that we do not limit ourselves to offer the authors’ main findings, but also provide some salient sociocultural aspects that we believe enrich the pictures of these (agro)pastoral lives. Overall, we aim at bringing more attention to the study of plant domestication in pastoral contexts of the New World, to animal agency in such processes, and to the cultural aspects that permeate them; that is, the links of biological and cultural diversity as a whole.

## Old World Livestock Species in the Americas

The myriad of exotic biota, technologies, and management practices that were introduced during the Spaniard Conquest (15th century) resulted in competition and the eventual fusion of the native and alien agricultural systems. The new hybrid agro-pastoral landscapes that emerged (Butzer, 1988; Gade, 1992; Whitmore and Turner, 1992; Sluyter, 1996) have been considered more or less ecologically sustainable during both, the centuries of colonial occupation (e.g., Fisher et al., 2003), and in modern times (e.g., Toledo and Barrera-Bassols, 2005; Ford and Nigh, 2009). Peoples have learned how to use introduced technologies, including animals, and through them resist further dispossession. Moreover, some socioecological systems, like the modern world-renowned Maya forests, cannot be understood without the combined effects of people and Old World livestock (Campbell et al., 2008).

All across the continent, a multitude of identities evolved from livelihoods dependent on livestock husbandry, among them the popular North American *cowboys*, the Mexican *vaqueros*, or the Argentinean *gauchos* (Camou-Healy, 1998; Narchi et al., 2015). As broadly as cattle ranching, although less known, transhumance of cattle, sheep and goats also emerged at continental scale, from Northern Mexico up to the Patagonia (Thornton et al., 2003). Whether permanent or transhumant, after five centuries of coevolution, one can expect that indigenous societies that fully embraced livestock raising have co-constructed particular niches with them. While some research exists regarding the different roles that livestock have had in the Americas’ post-conquest development (e.g., Melville, 1994; Camou-Healy, 1998; Narchi et al., 2015), few have approached them as protagonists of biocultural diversity persistence and evolution, as well as biodiversity promotion (e.g., Perezgrovas, 2004; Lanari et al., 2005).

### Navajo Livestock and Plant Domestication

Kuznar (2001) carried out a study of Navajo anthropogenic habitats, including their livestock’s corrals, and determined that these are one more instance where human–animal–plant ecologies result in the establishment and growth of plants which are also useful to humans. The Navajo live in a reservation covering northeast Arizona, northwest New Mexico and portions of Utah (United States), where the pastoral environments consist of pinyon-juniper forests and sagebrush steppes from 1,875 masl to 2,500 masl. Herds of sheep, cattle, and horses have been part of Navajo life since the eighteen century, but became the core of their livelihoods as transhumance developed about a century later. Among the reasons to keep animals, Navajos argue that livestock raising is the only way to keep traditional land-use rights and that livestock are of social as well as religious relevance.

The author studied a sample of corrals for all animals (sheep, horses, cows), registering the plant species, as well as their number, growing in and around them. From a total of forty plant species found, twenty-eight were pioneer annuals prone to be transported to the corrals by livestock. Using certain criteria of species density (# individual plants/m^2^), he identified that twelve of such pioneer species were the most common in corrals but also frequently found in other anthropogenic environments such as abandoned cornfields, habitation areas or roadsides (**Table 1**). Eight of those species were traditional foods that would be harvested in abandoned corrals: beeweed, *Cleome lutea*; goosefoot, *Chenopodium album*; tansy mustard, *Descurainia pinnata*; tumble mustard, *Sisymbrium altissimum*; knotweed, *Polygonum aviculare*; *Amaranthus graecizans*; tumbleweed, *Salsola iberica*; and *Halogeton glomeratus.* These two latter are chenopods of recent introduction and closely related to the indigenous goosefoot, *Chenopodium album*. The four remaining species were reported to be of health or ceremonial or utility: cheeses, *Malva neglecta*, is used as a lotion on injuries; skyrocket gilia, *Gilia aggregate*, is used as a remedy for stomach problems and as a luck preparation for hunters; filaree, *Erodium cicutarium* is used on prayersticks in Navajo ceremonies as well as a sheep forage; and, finally, the wild tobacco, *Nicotiana attenuate*, is a multipurpose ceremonial species used, for example, to smoke over the livestock everyday as a protective ritual (Kuznar, 2001).

The ecological succession sequences that follow corral installations by the Navajo were also described by the author. The process by which useful weedy annuals emerge starts when people modify the land by clearing an area for building a corral. Then, corrals are used for 5–10 years, when manure accumulates, surrounding trees disappear (as a result of animal wastes, they die within a few years), and there is a general change in soil conditions in and around them (organic matter accumulation, moisture raise) that are favorable for weeds to start colonizing these sites; eventually, the plants are either collected by people or eaten by animals, being observable only seasonally, when the corrals are not in use. Lastly, the corrals are deserted and pioneer species take over these disturbed habitats completely. Under historic Navajo land use, further successional stages would have followed, including the return of different species that would have become both habitat and food for wild animal species such as cottontail rabbit (*Sylvilagus* spp.), jack rabbit (*Lepus* spp.), or mule deer (*Odocoileus hemionus*). In the end, pinyon-juniper communities would grow again and provide both habitat for larger game species as well as grazing environments for livestock. Such cycle is expected to take about 20–40 years.

Finally, the mutualistic relationships among humans, livestock and plants appear to be coded in traditional knowledge: Navajo belief that the Holy People (*diyini diné’é* or *haashch’éé diné’é*) gave them livestock to use, so they have to respect such gift. Animal care is the form by which people show respect and, in retribution, the Holy People send rain and food for them; when livestock flourish, so do people. According to this religious perspective on herding, there is interdependence of human, animal and plants lives. Sheep bear the life-giving fluids and saturate the earth with them, which in turn are taken by the plants when they grow; moreover, the plants eventually become sheep again. Sheep are also the carriers of valuable plants, saving them in their hooves and eyelids, and providing people with them.

### Rarámuri Livestock and Plant Domestication

The pre-Hispanic Rarámuris inhabited the extensive plains and highlands of the modern state of Chihuahua (Northern Mexico), where a failed crop would have been overcome with hunting, fishing and gathering upon the abundant and rich flora and fauna of the region. First Spaniard, and then *mestizo* continuous colonization of the best lands for agriculture forced them to stay mainly in the highlands, where agriculture is more difficult given the harsh biophysical conditions of mountains and canyons (Granados, 2006). Today’s Rarámuri identity is still based on the making of *milpa*, a polycropping system where mainly squash, beans and chili accompany the core species, maize (*Zea mays*). They are also well-known because of their distinctively high mobility; they change their residence in order to cultivate dispersed fields and, if they own livestock, move to canyons in order to protect them from the coldest weather in mountains. Actually, the classical ethnographic descriptions of Rarámuri life depicted them as mixed agro-pastoralists (Hard and Merrill, 1992; Granados, 2006).

The Rarámuris differentiate the realm of cultivated lands, the *wasachí*, from the one of wild lands or devil’s lands and the sierra in general, the *kawichí*. However, no strict divide between them exists, for they see both landscapes as a continuum with gradients of humanity within: the ‘least human’ extreme would be the *mestizo* towns; the ‘most human’ would be the *wasachí*; finally, the *kawichí* would occupy an intermediate position, populated by wildlife, supernatural beings, and God’s crops (i.e., the pines are his ‘maize’) (Wyndham, 2009, p. 282). More than 350 plant species are edible in the sierra, and the most important for the Rarámuris are approximately 120 species of *quelites* (Mexican Spanish) or *kiribá* (Rarámuri form; Wyndham, 2009), a fundamental component of people’s diet, and pertaining to both the *wasachí* and the *kawichí.* This is so because they are wild plants, generally herbaceous, however, are strongly related to human-disturbed habitats such as cultivated fields, field-fence margins, dwelling sites, animals’ corrals, and trailsides (Bye, 1981; Wyndham, 2009). There is a dynamic and constant interaction between the *wasachí* and *kawichí* as the daily exchange of nutrients and seeds occur while cattle, sheep, and goats graze in the rangelands and return to their corrals (Bye, 1979, 1981).

Bye (1979) documented phenotypic changes in plant species growing in corrals where Rarámuris pen their animals. He described how people collect wild mustard plants (*Brassica campestris*) in the *kawichí*, but they also plant them in the *wasachí*, specifically in goats and sheep corrals, where the plants thrive; then, those specimens with larger leaves are selected by people (**Table 1**). Livestock species also modify the landscape incidentally when their owners keep certain trees because they serve to store livestock fodder and to give shade, for instance (Wyndham, 2009). The cultural relevance of livestock species is also attested by the year-round activity of weaving with sheep’s wool (wide belts and blankets of intricate designs) and by their participation in ritual life, since they are not killed for food but for religious sacrifices only (Pintado, 2004; Granados, 2006). The integration of cattle, sheep and goats to the Rarámuri *milpa* system helped to make its endurance less strenuous. The aggregated consequences of intentional and unintended actions of people and their animals, for sure have influenced the landscapes and biological diversity of this region of the Sierra Madre Occidental (Wyndham, 2009).

### Aymara Livestock and Plant Domestication

In the Andes of Southern Peru, Kuznar (1993) described the mutualism between humans, livestock and plants happening in the so-called *sierra alta* and *puna* habitats. Besides, he developed a hypothesis of *quinoa* domestication (*Chenopodium quinoa*) based on observations of the current interactions among other two chenopods and livestock species in these habitats. The author also highlighted the salient outcomes that anthropogenic as well as animal modified environments have upon the distribution and concentration of those two and other plant species.

In the Andean *sierra alta*, the deep valleys and abrupt terrains that stretch out from 2,500 to 3,800 masl support Aymara agro-pastoralists who cultivate potato and other tubers (*oca, ulluco*), *quinoa*, barley, as well as other vegetables. Above 3,800 masl, the *puna* sustain a more exclusively pastoralist agriculture of mixed herds of alpacas, llamas, sheep, goats and cows. Besides, manure has been fundamental for local agriculture since organic matter is a restricted in these areas.

Transhumance is practiced as herders move from lower zones (2,500–3,200 masl) during the wet season, to higher ones (3,200–3,800 masl) during the dry season. In these systems, the author identified the main forage plants used by livestock in valleys located at 2,500–3,800 masl. Among these, two species of *Chenopodium* were among the most preferred plants not only by goats but also by sheep, cows, and the indigenous llamas, so a prehistoric mutualism between llamas and these forage plants, the author argues, is possible. Recognizing the predilection of goats for such plants, Aymara herders name one of them as *quinoa chivo* (goat *quinoa*); the other was identified as *Chenopodium petiolare.* Stem and leaves’ portions as well as seeds of these and other species are commonly found in the animals’ fur and dung. Other mutualisms take place with *Dunalia brachycantha*, favored by goats, *Mutisia acuminata* (*chinchelcoma*), also favored by goats, and *Cantua candelilla* (*cachicana*) preferred by both goats and cows (**Table 1**). When their frequencies in abandoned campsites *vs.* wildlands were measured (# plants/ha), the author found a much higher representation of these plants than expected at the pastoral sites, given their distribution throughout the valley. Only for *Chenopodium* spp., he found a 117 times higher abundance in campsites than in the rest of the area. Moreover, the two communities in which chenopods are found at largest quantities are also the most heavily grazed.

Since herds are left to graze at large during the day and returned to corrals at night, the mutualism occurs while animals, in choosing their favorite food, disseminate their seeds as their feces accumulate in corrals, but also over the entire rangelands. The corrals, however, would be the most fertile and humid locations of the area where plants would thrive. Because chenopods are hardy, opportunistic pioneer species, and animals eat everything but their stem and roots, the author proposes that they would endure the animals’ grazing when grown within and near corrals; then, chenopods would grow new spurts and produce seeds hastily. Additionally, shrubs that inhibit chenopods growth are continuously pruned by goats, so they would also be setting the habitat for these plants by limiting the growth of their competitors (Kuznar, 1993).

The results of this study strongly suggest that livestock-*Chenopodium* relations are responsible for the geographic association of these plants with the human-livestock habitation sites. According to the author, since the wild ancestor of *quinoa* would have had similar habits to the wild varieties studied by him, its domestication process would have started with individuals establishing themselves in people’s camps because of their more favorable characteristics, and concentrating there out of animal transportation of their seeds via pastoralism; eventually, the high concentration of this chenopod in pastoral sites and its broad utilization, would have led also to cultivation and selection practices by Aymara herders. Chronologically, first human-animal relations would have been in progress, later the plant would have joined such herding ecologies, which is in agreement with the archeological record (herding appears at 6,000–3,400 years ago, while domestic quinoa emerges about 3,000 years ago). Kuznar concludes asserting that just as plant communities’ composition in the past have been modified by llama and alpaca grazing (using palynological analysis) during millennia, correspondingly, modern herds dominated by goats began similar routes since their arrival to the Andes (see also Marsh, 2015; Fritz et al., 2017).

### Mapuche Transhumance in the Patagonian Monte

Ladio and Lozada (2009) reviewed the contemporary use of wild edible plants in relation with cattle transhumance in the so-called *monte* region of Argentina (**Table 2**). Indigenous peoples such as the Huarpes, Calchaquies, Puelches, Pehuenches, Ranqueles, Tehuelches, and Mapuches, whose livelihoods were based on agriculture, wild plant gathering, and hunting, were devastated almost totally with the Spaniard colonization. Eventually, they lost their lands and had to move to the most remote and unproductive regions. Mapuche people, in particular, began to dwell the *monte* areas in the seventeenth century and adopted the European livestock species. Today, they still depend on the plant diversity of the *monte* habitats in order to obtain food, medicines, products for selling, materials for construction and domestic tools, or ceremonial aims. Transhumance is practiced during summer and winter seasons, and it is locally referred as *veranada*. Pastoralists and their herds move from the *monte* or *travesía* environments to Andean forests of *Araucaria araucana* (Pehuén forests), collecting edible seeds and plants while traveling and making use of the rangelands. From a comparative study, they also found that people practicing transhumance augment the richness and quantity of the plants they use *vs.* those who do not travel (Ladio and Lozada, 2004).

On separate investigations, Lanari et al. (2005) have been studying related and overlapping Argentinian socioecological systems in the northern province of Neuquén (**Table 2**). Their focus, however, is on ethnozoology instead of ethnobotany. As product of their research in areas of Mapuche and Tehuelche cultural roots, they have worked with local communities of goat breeders, whose livelihood depends on a transhumant way of life. These *crianceros* or goat breeders have exercised specific selection criteria upon their animals for generations, actually giving origin to a breed named the *Neuquén criollo* goat, a national genetic resource.

Presumably, the human and animal-made environments such as habitation areas, corrals, trails, and so on, of these pastoral niches would be good for different plant species to establish themselves, be consumed by both people and livestock, and even be planted and selected according to specific criteria. As we saw in the Navajo, Rarámuri, and Aymara socioecological systems, weeds such as chenopods are potential and actual colonizers of such habitats, some are edible for people and animals, and some are cultivated. Ladio and Lozada (2009) reported four chenopods among the most used species of the Patagonian *monte*: *Chenopodium oblanceolatum, Atriplex lampa, Suaeda divaricata*, and *Allenrolfea vaginata*. For their part, Castillo and Ladio (2017) recorded fourteen plant species of ethnoveterinary use by others communities of *crianceros*, also in Argentina (**Table 2**). Given the utility of these species, in some cases for people as well as animals, they represent a potential source of additional human management (Casas et al., 2007; Clement, 2014) and further human–animal–plant mutualisms that remain to be investigated.

### Tzotzil Sheep and Plant Domestication

As already introduced when talking about Rarámuri people, in Mexico most contemporary peasants still have a way of life that revolves around maize cultivation. Cultures have given identity to *milpa* and vice versa as farmers, through their experience in their various habitats, have developed equally diverse kinds of this production system. As a proxy of *milpa* diversity, consider that roughly 60 languages are still spoken in Mexican territory, which is one of the most ecologically and culturally diverse countries not only in the continent but in the world (Boege, 2008). The significance of maize and *milpa* goes beyond their nutritional and economic value to people, as the following statements evidence: “to abandon one’s *milpa* is to forsake the very roots of life” (of the Yucatan Mayas; Redfield and Villa-Rojas, 1934); “[…] the making of *milpa* is the central, most sacred act, one which binds together the family, the community, the universe” (of the highland Mayas; Nigh, 1976) (*apud* Alcorn and Toledo, 1998, p. 233).

Tzotzil people live in the Chiapas Highlands, a mountainous area at approximately 2,200 masl. They speak their own language and dress in traditional garments made from sheep’s wool. According to tradition, men take care of *milpa* cultivation while women are responsible for the family, which includes sheep (**Table 2**). Small flocks of about a dozen animals can comprise more than a third of a family’s income (through commercialization of animals, wool, handicrafts and woolen garments, as well as manure). They cannot be killed nor eaten for, under Tzotzil cosmovision, sheep are the sacred animals that accompany the Holy Patron of people, Saint John the Baptist. Today, their identity is recognized not only as an element of Tzotzil culture, but also according to genetic criteria (similar to the *Neuquén criollo* goat) (Gómez et al., 2014). This Old World species is also responsible for a specific kind of soil documented by ethnopedology. Soils are essential for rain fed local agriculture and as such, Tzotzil farmers classify and recognize those following different criteria. The ‘gray soils’ develop when sheep manure is added to the ‘yellow silty soils’ that are afterward used for cultivation, and only occur in the communities practicing agropastoralism, where sheep graze within karstic zones (Bandeira et al., 2002). Even though no specific study on plant domestication dependent on sheep has been carried out for the Tzotzil socioecological systems, the pervasive use of herbal medicines for their health and productivity is well known (Perezgrovas et al., 2014). Importantly, and just as in the case of soil or any other natural resource, the management of these plants happens within the context of indigenous knowledge system and cosmovision. Tzotzil shepherdesses know precisely what plant, combination of species, and/or rituals are required for the treatment of common sheep diseases. They gather them in the wild or near habitation spaces and some of them are also used for human treatments (Geerlings and Perezgrovas, 2014; Perezgrovas et al., 2014). Whether any of these or other species are under incipient management other than simply gathering (Casas et al., 2007; Clement, 2014), or specifically associated with Tzotzil herding ecologies, remains to be investigated. However, and alike other indigenous landscapes in America, Tzotzil *milpas*, home gardens, orchards, rangelands, fallow fields, forests in various stages of ecological succession, among others, constitute potential arenas for such an outcome.

## Native Animal Species of the Americas

### Camelid Husbandry and the Domestication of Andean Landscapes

Camelid agropastoralism has been historically complementary to hunting and gathering in South American since its origins, and continues to be characterized as a remarkable resilient system capable of responding to past and current climate changes (Marsh, 2015). Though Kuznar (1993) reported the abovementioned case study of plant domestication under the context of pastoralism of alien livestock species, this could potentially be also applied for American camelids (llamas *Lama glama*, and alpacas *Vicugna pacos*) (**Table 1**). To our knowledge, however, his work has been the only one of the kind in South America.

Given its weedy tendencies and role as forage for camelids, for chenopods such as *quinoa*, it has been proposed after Kuznar (1993), recently and more than once (Pearsall, 2008; Marsh, 2015; Fritz et al., 2017), that in prehistoric times such wild species proliferated in human-camelid environments, eventually resulting in the cultivation of the ancestors of *quinoa* and other species. The hypothesis of simultaneous management of camelids and certain plant species is partially supported by both archaeozoological and archaeobotanical research in the Andes (i.e., earlier presence of domestic camelids than plants; morphological changes of animals together with greater abundance of Chenopodiaceae-Amaranthaceae and *Pennisetum*, both signaling human management) (Marsh, 2015; Yacobaccio and Vilá, 2016).

### Reindeer Husbandry and the Domestication of Arctic Landscapes

Since prehistoric times, reindeer or caribou (*Rangifer tarandus*) populations have shared the Arctic territories with different indigenous peoples, providing them with hunting, milking and transportation goods. Both wild and domestic engagements between humans and reindeer happen at particular points, not at random but in places with predictable characteristics according to people’s and animals’ knowledge of the land (Baskin, 2003; Andrews et al., 2012; Anderson et al., 2014, 2017).

Anderson et al. (2014), using different archeological and ethnographic data, investigated the signals that human-reindeer ecologies can leave on the landscape (**Table 2**). Based on archival and ethnographic information, these authors were able to locate what Evenki pastoralists (Siberia) refer to as ‘good places’ or ‘places that suggest themselves’ for they are considered to bear agency. These areas are rangelands or meadows that constitute important forest clearings used as ‘stopping areas’ for both Evenkis and reindeer given their aptitude for satisfying the needs of both. There, people make fire, place their tents, practice agriculture, and watch the herds. Their sampling results (i.e., pollen, plants, fungal spores, soil), on the other hand, allowed them to document some of the historical and contemporary activities of Evenki pastoralists and their reindeer such as reindeer penning, maintenance of rangelands, or formation of vegetation patches. This was achieved by interpreting the species composition of plant communities, which vary according to the trampling intensity due to human-reindeer presence, allowing their classification in functional zones such as those for dwelling, milking and grazing. The authors proposed a model of plant succession where the taiga forest transitions to a meadow community, where signals of cereal agriculture as well as ‘some clear markers of the effects of animal agency such as *Polygonum aviculare*, Chenopodiaceae, and Poaceae’ could be detected; from here, the forest eventually recovers. Based on their botanical analyses, but also on the animals’ behavior, the authors conclude that reindeer create particular sets of plant communities that may be markers of East Siberian Rangifer agency (Anderson et al., 2014). This so happens not only via the accumulation of their manure, which modifies the soil, but also through the exposition of the permafrost when they are feeding upon the moss cover, inducing melting that creates small lenses of water. The estimates of these land uses occurrence were of about 700 years, that is, go back to the 14th century. Importantly, people recognize reindeer autonomy and their ability to choose the best places to find food and avoid insects and predators, so they follow the herds while animals search for the aforementioned ‘good places’ during their seasonal movements.

Reindeer feeding and habitat selection behavior, as well as their effects on the plant communities are also known for Canadian regions at both recent (e.g., Bodreau and Payette, 2004; Johnson et al., 2004) and historical times (Andrews et al., 2012). However, such data have been acquired on the basis of indigenous hunters’ ethnographies, as well as ecological and archeological analyses. To our knowledge, there has not been an explicit examination of the effects that reindeer husbandry can have on the landscape; i.e., the plant communities, as Anderson et al. (2014) have done in Siberia.

In Northwest Canada, Andrews et al. (2012) doing archeological research on the relation between reindeer and their habitat, and that of the latter with human hunting, have described particular places in the landscapes where animals and people engage both historically and currently (**Table 2**). The ice patches selected by reindeer in these Canadian alpine forests occur on north- and northeast-facing slopes and vary according to other environmental factors such as snow depth and hardness, and the presence of forage. These areas tend to melt and be surrounded by reindeer manure. The dating of reindeer dung layers in such areas result in associations of about 5,000 years before present. The authors also report that contemporary Shúhtagot’ine hunters still make use of ice patches to kill reindeer according to detailed knowledge of local ecology, but also to a cosmovision where the landscape is sacred. Like other societies of the circumpolar territories, the Shúhtagot’ine believe that the world is instilled with ‘will and purpose’ or agency. Under such vision, animals are other-than-human persons that, together with other spiritual beings, share the land with them.

These examples illustrate how humans, reindeer and plant communities interact in their environmental niches, both in hunting and pastoral ecologies. However, it would be interesting to deepen and refine the knowledge of the most northern pastoral niches of the continent by means of ethnobotanical surveys (for the Gwich’in people of Canada, for instance, but also for other reindeer herding ecologies of the circumpolar Arctic as well, see Anderson et al., 2017; **Table 2**). Possibly, among the diverse possibilities that human–reindeer–plant interactions can take, there are species in the flora reported by Anderson et al. (2014) in Siberia, or those known for Canada (e.g., Uprety et al., 2012), that are distinctively managed by pastoralists. In Fennoscandia, for example, Rautio et al. (2016) recorded how Sami people have historically managed *Angelica archangelica*, one of the most important plants used and managed not only as food and medicine, but also and interestingly, as preservative for reindeer milk (**Table 2**). Besides plants of dietary use, others of ethnoveterinary qualities may be discovered.

## Discussion

Under the most conservative views of species domestication and the transition from foraging to farming, only a few places and species dominate the research arena. This is so because the idea of ‘centers of domestication’ is associated with the concept of founder agricultural packages. These accounts are often linked to sharp distinctions between artificial categories such as ‘wild’ *vs.* ‘domestic’ or ‘foraging’ *vs*. ‘farming.’ In addition, the bulk of work on species domestication seems to always approach humans and target species of either plants or animals as if these organisms only lived in two-way partnerships with humans. Moreover, people have the leading role in such partnerships, with animals and plants being more or less passive objects responding to human will.

In contrast to these narratives, what we intend here is to underscore animal agency –of livestock species– in human niche construction –the pastoral niche. In addition, we also intend to further bear on alternative reflections of domestication as a composite of human behaviors and relationships with other species whereby humans modify their landscapes, as opposed to simply consider them as end results that can always be somewhat measured –at both landscape and populations levels, both in the past and present (Terrell et al., 2003; Bellwood et al., 2007; Zeder, 2015). Under this vision, countless additional species and landscapes can be considered ‘domesticated’ and the picture provided by the so-called ‘centers’ or ‘cradles’ of domestication changes considerably. This is of the highest relevance in terms of food production and sustainability. For instance, only 100 plant species account for 90% of the present world’s food production, while approximately three to five thousand species were once used as food in North America alone. Therefore, there are many underutilized species that are yet to be assessed not only for bioprospective ends but also as means for enhancing agrobiodiversity, managing climate risk, and improving rural livelihoods (Meyer et al., 2012; Lira et al., 2016).

In the American continent, domestication centers traditionally refer to areas of Eastern North America, Mesoamerica, the Andes, and the Amazon (Smith, 2006; Casas et al., 2007; Pearsall, 2008; Stahl, 2008; DeClerck et al., 2010; Piperno, 2011; Clement, 2014), even though there is also a wealth of knowledge about landscape, plant and animal domestication in the most northern regions by the Arctic, Northwest Coast, Prairie, Neutral or Huron cultures (Berkes and Davidson-Hunt, 2006; Turner et al., 2013; Thornton et al., 2015; Anderson et al., 2017). From this work and the case studies we provide, we can observe that people manage landscapes co-habited and co-created by animals and plants interacting among them and with the rest of the denizens of their shared *domus*, however, small or ample (e.g., intensive *vs.* extensive farming), perpetual or ephemeral (e.g., sedentary *vs*. mobile) this might be. In other words, domestication is a multispecies endeavor, one that sometimes is untraceable or barely visible across time and space (like salmon and herring streams used by fishermen, or short-lived reindeer husbandry), but not less important because of that. Moreover, the issues of human exceptionalism and non-human agency in domestication processes become more visible since ethnographic evidence shows us that human beings, contrary to dominant conceptions, do not always guide animal behavior, but carefully observe and commonly follow the lead of other animals. The traditional herding ecologies of the Americas are lively examples of this pattern, with indigenous (agro)pastoralists acknowledging the animals’ role in their management decisions.

In all the cases reviewed here, it can be seen how the pastoral niche is immersed in the complex knowledge-practice-beliefs of indigenous people, and how reindeer, cattle, sheep or goats are ‘ecosystem engineers’ or ‘landscape architects’ just as humans are (Zeder, 2016; Anderson et al., 2017). New World pastoralist societies have continuously been adding species to the humanity’s portfolio of useful plants not only by themselves, but many times aided by their animals in different ways. The species of weedy nature are particularly salient among these (**Table 1**). Remarkably, this is true around the world for both prehistoric as well as modern domesticates such as *Amaranthus, Chenopodium*, sunflower (*Helianthus annum*), knotweed (*Polygonum erectum*), or the cherry tomato (*Lycopersicum* spp.), among others, in the New World (reviewed by Kuznar, 1993), and the wild forms of species as important as rye (*Secale* spp.), oat (*Avena* spp.), barley (*Hordeum* spp.), einkorn wheat (*Triticum monococcum*), hemp (*Cannabis sativa*), melon (*Cucumis trigonus*), or carrot (*Daucus carota*) also favoring disturbed habitats in the Old World (Vavilov, 1926 in Kuznar, 2001; see also Abbo et al., 2005).

Although some domestication literature does mention that plant and animal domestication influenced each other, not much detail is given on how this happened (Zeder, 2011; Larson et al., 2014; Vigne, 2015; Bar-Yosef, 2016). We think that this is a place/time specific issue, so that it could have happened sometimes earlier, later, or simultaneously, and is on a case by case occurrence that human-animal relationships leading to plant domestication (or the other way around) can be hypothesized. For instance, for the first ‘primary’ species of the Old World agriculture including the lentil (*Lens culinaris*), pea (*Pisum sativum*), chickpea (*Cicer arietinum*), among others, some authors reject the ‘dump-heap’ or ‘camp-following’ species hypothesis (Abbo et al., 2005), while for the ‘secondary’ crops it is not discarded (Abbo et al., 2005), and it has been widely advocated for the initial domestication of different New World species (Kuznar, 1993, 2001). Likewise, for the specific case of the Near-Eastern wild caprines, it has been proposed that some cultivation activity was destined for baiting during the initial steps in the domestication of such herbivores (Svizzero, 2016). We believe that domestication research is an endless enterprise of documenting the diversity of organisms that were/are under domestication, as well as the ever evolving environmental and cultural contexts where such processes occur. We think the ethnographic studies reviewed here add detail to this subject.

Herd animals bring entire landscapes and their plant species to human attention while domesticating their own environments. While many plant species had been for certain in use by Native Americans in pre-Columbian times, countless others became new additions to their useful flora portfolio indirectly, that is, through consumption of Old World livestock species or their products, or directly, under the umbrella of human-animal relations as food (like Mexican *quelites*), medicine (like ethnoveterinary treatments), or for ritual purposes (like those for amulets, charms, and so on). The livelihoods based upon the immigrant herds of cows, goats, or sheep, just to mention the most conspicuous species, have had paramount consequences for the cultural identities and biological diversity of the continent (i.e., the biocultural diversity). Yet, to our knowledge, they have only been meagrely and superficially approached from a domestication perspective (with the exemption of the studies presented here). By the same token, it seems right to recognize that the forebears of these livestock species could have had similar effects in the Old World at the eve of agriculture (Langlie et al., 2014; McClure, 2015).

As explained by Price and Bar-Yosef (2011, pp. 163–164) on the origins of agriculture, each participant contributes with his or her own expertise but also with biases; these offer insight but can also work as blinders, which can be obstacles to broader synthesis. Perhaps it is because herbivores such as sheep or goats are still considered alien to these lands, or out of ‘ancientism’ in domestication studies (similar to ‘recentism’ in environmental history studies; see Sluyter, 2005), and for sure because scholarship is divided between botany and zoology, that is clear that American pastoralists have not received as much attention as the attractive Old World pastoralists with respect to how they and their animals domesticate their landscapes. Today, they still inhabit large and ecologically diverse areas of the continent. We encourage scholars to pay closer attention to these herding ecologies. From such research, we would expect not only the enrichment and refinement of our understanding of domestication instances and processes, but also further appreciation of the holistic and detailed knowledge people have of their environments and how they change through time. Future work should focus on integrating scientific as well as traditional knowledge frameworks of rangeland management if sustainability is to be achieved via bioculturally sound options (e.g., Risvoll and Hovelsrud, 2016).

## Conclusion

Domestication studies are critical for understanding the effects that ancient societies had in modern landscapes and biodiversity, and how they continue to do so. They provide us with information of the uses, domestication history, and phylogenetic relationships of a range of species. This knowledge can help in amending and improving our current frameworks of governance, conservation, and restoration of useful species and habitats. Similarly, it could contribute to improve food security as well as biocultural diversity altogether.

Throughout our review, we identified empirical work on the question of ‘how’ domestication at both landscape and plant populations levels can occur in the multispecific/multiagentic pastoral niches. Such work includes both archeological (e.g., Langlie et al., 2014; Marsh, 2015; Fritz et al., 2017) as well ecological evidence (**Table 1**). We note that, although reliable chronologies for either the emergence of pastoralism and/or the morphological distinction of domestic species more generally, often remain elusive, this is due to the very nature of the archeological record. This kind of evidence is fragmented, always increasing, and continuously being revaluated (e.g., Snir et al., 2015). Thus, in the absence of perfect and complete data, any reconstruction of past events can only be qualified as more or less likely. This is why we have also tried to show how the ethnographic present can illuminate the past by focusing on contemporary animal and plant management practices. Rephrasing fellow ethnobiologists, ancient humans and our primate relatives must have keenly observed their surrounding plant communities and the feeding behavior of animals; from such scrutiny, they must have learned about the ecology of those communities, about edible plants, how to harvest them, and began experimenting with cultivation (Turner et al., 2011, p. 213). We also brought attention to more recent, related research that is yet to be expanded and integrated under similar frameworks with potentially fruitful results (**Table 2**). Analogous work could be carried out not only in the Americas, but wherever animals, with their intelligent adaptability, join plants and humans in their mutual niche construction activities. We hope that the examples appraised here suffice to bring more attention to contemporary pastoralists as plant managers, animals as agents in human–plant interactions, and domestication as a behavioral complex and multispecies process that is as important in the present or future, as it was in the past.

## Author Contributions

PL-N developed the initial concept and outline. DS-F and JV improved the proposal and the manuscript. DS-F edited the manuscript. All authors read and approved the final manuscript.

## Conflict of Interest Statement

The authors declare that the research was conducted in the absence of any commercial or financial relationships that could be construed as a potential conflict of interest. The reviewer GB-G and handling Editor declared their shared affiliation.

## References

[B1] Abbo S. Gopher A. (2017). Near Eastern plant domestication: a history of thought. 22 491–511. 10.1016/j.tplants.2017.03.010 28434795

[B2] Abbo S. Gopher A. Rubin B. Lev-Yadun S. (2005). On the origin of Near Eastern founder crops and the ‘dump-heap hypothesis’. 52 491–495. 10.1007/s10722-004-7069-x

[B3] Alcorn J. B. Toledo V. M. (1998). “Resilient resource management in Mexico’s forest ecosystems: the contribution of property rights,” in eds Berkes F. Folke C. (Cambridge: Cambridge University Press) 216–249.

[B4] Anderson D. G. Ineshin E. M. Kulagina N. V. Lavento M. Vinkovskaya O. P. (2014). Landscape agency and Evenki-Iakut reindeer husbandry along the Zhuia River, Eastern Siberia. 42 249–266. 10.1007/s10745-013-9632-6

[B5] Anderson D. G. Laurens J. P. Schroer S. A. Wishart R. P. (2017). Architectures of domestication: on emplacing human-animal relations in the North. 23 398–416. 10.1111/1467-9655.12613_1

[B6] Andrews T. F. MacKay G. Andrew L. Stephenson W. Barker A. Alix C. (2012). Alpine Ice Patches and Shúhtagot’ine Land Use in the Mackenzie and Selwyn Mountains, Northwest Territories, Canada. 65(Suppl. 1) 22–42. 10.14430/arctic4183

[B7] Bandeira F. P. López J. Toledo V. M. (2002). Tzotzil Maya ethnoecology: landscape perception and management as basis for coffee agroforest design. 22 247–272.

[B8] Bar-Yosef O. (2016). “Multiple origins of agriculture in Eurasia and Africa,” in eds Tibayrenc M. Ayala F. J. (Amsterdam: Academic) 297–331.

[B9] Baskin L. M. (2003). River crossings as principal points of human/reindeer relationship in Eurasia. 23 37–40. 10.7557/2.23.5.1653

[B10] Bellwood P. Gamble C. Le Blanc S. A. Pluciennik M. Richards M. Terrell J. E. (2007). First farmers: the origins of agricultural societies. 17 87–109. 10.1017/S0959774307000078

[B11] Berkes F. (2008). New York, NY: Routledge.

[B12] Berkes F. Davidson-Hunt I. J. (2006). Biodiversity, traditional management systems, and cultural landscapes: examples from the boreal forest of Canada. 187 35–47. 10.1111/j.1468-2451.2006.00605.x

[B13] Bodreau S. Payette S. (2004). Caribou-induced changes in species dominance of lichen woodlands: an analysis of plant remains. 91 422–429. 10.3732/ajb.91.3.422 21653398

[B14] Boege E. (2008). Mexico: INAH.

[B15] Bowles S. Choi J.-K. (2013). the co-evolution of farming and private property in the early Holocene. 110 8830–8835. 10.1073/pnas.1212149110 23671111 PMC3670368

[B16] Butzer K. W. (1988). Cattle and sheep from Old to New Spain: historical antecedents. 78 29–56. 10.1111/j.1467-8306.1988.tb00190.x

[B17] Bye R. A. (1979). Incipient domestication of mustards in Northwest Mexico. 44 237–258. 10.1080/00231940.1979.11757919

[B18] Bye R. A. (1981). Quelites – ethnoecology of edible greens – past, present and future. 1 109–123.

[B19] Camou-Healy E. (1998). Zamora: El Colegio de Michoacán.

[B20] Campbell D. G. Guittar J. Lowell K. S. (2008). Are colonial pastures the ancestors of the contemporary Maya forest? 28 278–289. 10.2993/0278-0771-28.2.278

[B21] Casas A. Otero-Arnaiz A. Pérez-Negrón E. Valiente-Banuet A. (2007). In situ management and domestication of plants in Mesoamerica. 100 1101–1115. 10.1093/aob/mcm126 17652338 PMC2759202

[B22] Castillo L. Ladio A. (2017). Traditional veterinary solutions for herders living in limited and changing conditions: a case study of “crianceros” of Central Northern Patagonia, Argentina. 145 90–101. 10.1016/j.jaridenv.2017.06.001

[B23] Clement C. R. (2014). “Crop domestication in the Amazon,” in ed. Selin H. (Dordrecht: Springer) 1475–1475.

[B24] DeClerck F. A. J. Chazdon R. Holl K. D. Milder J. C. Finegan B. Martínez-Salinas A. (2010). Biodiversity conservation in human-modified landscapes of Mesoamerica: past, present and future. 143 2301–2313. 10.1016/j.biocon.2010.03.026

[B25] Fisher C. T. Pollard H. P. Israde-Alcantara I. Garduño-Monroy V. H. Banerjee S. K. (2003). A reexamination of human-induced environmental change within the Lake Pátzcuaro Basin, Michoacán, Mexico. 100 4957–4962. 10.1073/pnas.0630493100 12671066 PMC153662

[B26] Ford A. Nigh R. (2009). Origins of the Maya forest garden: Maya resource management. 29 213–236. 10.2993/0278-0771-29.2.213

[B27] Fritz G. J. Bruno M. Langlie B. S. Smith B. D. Kistler L. (2017). “Cultigen chenopods in the Americas: a hemispherical perspective,” in eds Sayre M. P. Bruno M. C. (Berlin: Springer) 55–75. 10.1007/978-3-319-52849-6

[B28] Fuller D. Q. (2010). An emerging paradigm shift in the origins of agriculture. 17 1–12. 10.1111/j.1939-3466.2010.00010.x

[B29] Fuller D. Q. Denham T. Arroyo-Kalin M. Lucas L. Stevens C. J. Qin L. (2014). Convergent evolution and parallelism in plant domestication revealed by an expanding archaeological record. 111 6147–6152. 10.1073/pnas.1308937110 24753577 PMC4035951

[B30] Gade D. W. (1992). Landscape. System, and Identity in the Post-Conquest Andes. 82 460–477. 10.1111/j.1467-8306.1992.tb01970.x

[B31] Geerlings E. Perezgrovas R. (2014). “Tsa’nel, estudio etnoveterinario sobre prácticas de manejo y medicina tradicional realizadas por pastoras tzotziles,” in ed. Perezgrovas R. (Mexico: Instituto de Estudios Indígenas) 138–166.

[B32] Gómez T. H. Castro H. Perezgrovas R. (2014). “The real sheep of the Tzotzil shepherdesses,” in ed. Perezgrovas R. (Mexico: Instituto de Estudios Indígenas) 274–288.

[B33] Granados V. (2006). Chihuahua: Instituto Chihuahuense de Cultura.

[B34] Gremillion K. J. Barton L. Piperno D. R. (2014). Particularism and the retreat from theory in the archaeology of agricultural origins. 111 6171–6177. 10.1073/pnas.1308938110 24753601 PMC4035987

[B35] Hard R. J. Merrill W. L. (1992). Mobile agriculturalists and the emergence of sedentism: perspectives from Northern Mexico. 94 601–620. 10.1525/aa.1992.94.3.02a00040

[B36] Hayden B. (2009). The Proof is in the Pudding: feasting and the origins of domestication. 50 597–601. 10.1086/605110 20642144

[B37] Hildebrand E. A. (2009). The utility of Ethnobiology in agricultural origins research examples from Southwest Ethiopia. 50 693–697. 10.1086/605569

[B38] Huffman M. A. (2003). Animal self-medication and ethno-medicine: exploration and exploitation of the medicinal properties of plants. 62 371–381. 10.1079/PNS2003257 14506884

[B39] Johnson C. J. Parker K. L. Heard D. C. Seip D. R. (2004). Movements, foraging habits, and habitat use strategies of northern woodland caribou during winter: implications for forest practices in British Columbia. 5 22–35.

[B40] Kuznar L. A. (1993). Mutualism between Chenopodium, herd animals, and herders in the South Central Andes. 13 257–265. 10.2307/3673655

[B41] Kuznar L. A. (2001). Ecological mutualism in Navajo corrals: implications for Navajo environmental perceptions and human/plant coevolution. 57 17–39. 10.1086/jar.57.1.3630796

[B42] Ladio A. H. Lozada M. (2004). Summer cattle transhumance and wild edible plant gathering in a Mapuche community of NW Patagonia. 32 225–240. 10.1023/B:HUEC.0000019764.62185.99

[B43] Ladio A. H. Lozada M. (2009). Human ecology, ethnobotany and traditional practices in rural populations inhabiting the Monte region: resilience and ecological knowledge. 73 222–227. 10.1016/j.jaridenv.2008.02.006

[B44] Lanari M. R. Domingo E. Pérez M. J. Gallo L. (2005). Pastoral community selection and the genetic structure of a local goat breed in Patagonia. 37 31–42. 10.1017/S1014233900001942

[B45] Langlie B. S. Mueller N. G. Spengler R. N. Fritz G. J. (2014). Agricultural origins from the ground up: archaeological approaches to plant domestication. 101 1601–1617. 10.3732/ajb.1400145 25326610

[B46] Larson G. Burger J. (2013). A population genetics view of animal domestication. 29 197–205. 10.1016/j.tig.2013.01.003 23415592

[B47] Larson G. Piperno D. R. Allaby R. G. Purugganan M. D. Andersson L. Arroyo-Kalin M. (2014). Current perspectives and the future of domestication studies. 111 6139–6146. 10.1073/pnas.1323964111 24757054 PMC4035915

[B48] Lira R. Casas A. Blancas J. (2016). New York, NY: Springer 10.1007/978-1-4614-6669-7

[B49] Marsh E. J. (2015). The emergence of agropastoralism: accelerated ecocultural change on the Andean altiplano, 3540–3120 cal BP. 20 13–29. 10.1179/1749631414Y.0000000036

[B50] McClure S. B. (2015). The pastoral effect. Niche construction, domestic animals, and the spread of farming in Europe. 56 901–910. 10.1086/684102

[B51] Melville E. K. G. (1994). Cambridge: Cambridge University Press 10.1017/CBO9780511571091

[B52] Meyer R. S. DuVal A. E. Jensen H. R. (2012). Patterns and processes in crop domestication: an historical review and quantitative analysis of 203 global food crops. 196 29–48. 10.1111/j.1469-8137.2012.04253.x 22889076

[B53] Molnar Z. (2017). “I see the grass through the mouths of my animals” – Folk indicators of pasture plants used by traditional steppe herders. 37 522–541. 10.2993/0278-0771-37.3.522

[B54] Nabhan G. P. (2000). Interspecific relationships affecting endangered species recognized by O’Odham and Comcáac cultures. 10 1288–1295. 10.2307/2641284

[B55] Narchi N. E. Búrquez A. Trainer S. Rentería-Valencia R. F. (2015). Social constructs, identity, and the ecological consequences of Carne Asada. 57 305–336. 10.1353/jsw.2015.0013

[B56] Nigh R. B. (1976). Ph.D. dissertion, Stanford University Palo Alto, CA.

[B57] Pearsall D. M. (2008). “Plant domestication and the shift to agriculture in the Andes,” in eds Silverman H. Isbell W. H. (New York, NY: Springer) 105–120. 10.1007/978-0-387-79407-5

[B58] Perezgrovas R. (2004). 3rd Edn. San Cristóbal de Las Casas: Instituto de Estudios Indígenas.

[B59] Perezgrovas R. Pedraza P. Peralta M. (2014). “Plants and prayers. Animal healthcare by Indian shepherdesses,” in ed. Perezgrovas R. (Mexico: Instituto de Estudios Indígenas) 220–223.

[B60] Pintado A. P. (2004). “Tarahumaras,” in ed. Indigenas C. N. (Mexico: PNUD).

[B61] Piperno D. R. (2011). The origins of plant cultivation and domestication in the new world tropics patterns, process, and new developments. 52(Suppl.4) 453–470. 10.1086/659998

[B62] Price T. D. Bar-Yosef A. (2011). The origins of agriculture: new data, new ideas: an introduction to supplement 4. 52 163–174. 10.1086/659964

[B63] Rautio A. M. Axelsoon W. Östlund L. (2016). “They followed the power of the plant”: historical Sami harvest and traditional ecological knowledge (Tek) of Angelica archangelica in Northern Fennoscandia. 36 617–636. 10.2993/0278-0771-36.3.617

[B64] Redfield R. Villa-Rojas A. (1934). Washington: Carnegie Institution of Washington.

[B65] Risvoll C. Hovelsrud G. K. (2016). Pasture access and adaptive capacity in reindeer herding districts in Nordland, Northern Norway. 6 87–111. 10.1080/2154896X.2016.1173796

[B66] Shahack-Gross R. Finkelstein I. (2008). Subsistence practices in an arid environment: a geoarchaeological investigation in an Iron Age site, the Negev Highlands, Israel. 35 965–982. 10.1016/j.jas.2007.06.019

[B67] Sluyter A. (1996). The ecological origins and consequences of cattle ranching in sixteenth-century New Spain. 86 161–177. 10.2307/215954

[B68] Sluyter A. (2005). Recentism in environmental history on Latin America. 10 91–93.

[B69] Smith B. D. (2006). Eastern North America as an independent center of plant domestication. 103 12223–12228. 10.1073/pnas.0604335103 16894156 PMC1567861

[B70] Snir A. Nadel D. Groman-Yaroslavski I. Melamed Y. Sternberg M. Bar-Yosef O. (2015). The origin of cultivation and proto-weeds, long before Neolithic farming. 10:e0131422. 10.1371/journal.pone.0131422 26200895 PMC4511808

[B71] Stahl P. W. (2008). “Animal domestication in South America,” in eds Silverman H. Isbell W. H. (New York, NY: Springer) 121–131. 10.1007/978-0-387-74907-5_8

[B72] Svizzero S. (2016). Hunting strategies with cultivated plants as bait and the prey pathway to animal domestication. 2 53–68. 10.20431/2454-8677.0202007

[B73] Terrell J. E. Hart J. P. Barut S. Cellinese N. Curet A. Denham T. P. (2003). Domesticated landscapes: the subsistence ecology of plant and animal domestication. 10 323–368. 10.1023/B:JARM.0000005510.54214.57

[B74] Thornton P. K. Kruska R. L. Henninger N. (2003). Locating poor livestock keepers at the global level for research and development targeting. 20 311–322. 10.1016/S0264-8377(03)00034-6

[B75] Thornton T. Geur D. Kitka H. (2015). Cultivation of salmon and other marine resources on the Northwest Coast of North America. 43 189–199. 10.1007/s10745-015-9747-z

[B76] Toledo V. M. Barrera-Bassols N. (2005). Ethnoecology of the Yucatec Maya: Symbolism, Knowledge and Management of Natural Resources. 4 9–41. 10.1353/lag.2005.0021

[B77] Turner N. Łuczaj L. J. Migliorini P. (2011). Edible and tended wild plants, traditional ecological knowledge and agroecology. 30 198–225. 10.1080/07352689.2011.554492

[B78] Turner N. J. Lepofsky D. Deur D. (2013). Plant management systems of British Columbia’s first peoples. 179 107–133.

[B79] Uprety Y. Asselin H. Dhakal A. Julien N. (2012). Traditional use of medicinal plants in the boreal forest of Canada: review and perspectives. 8:7. 10.1186/1746-4269-8-7 22289509 PMC3316145

[B80] Vigne J. D. (2011). The origins of animal domestication and husbandry: a major change in the history of humanity and the biosphere. 334 171–181. 10.1016/j.crvi.2010.12.009 21377611

[B81] Vigne J. D. (2015). Early domestication and farming: what should we know or do for a better understanding? 50 123–150. 10.5252/az2015n2a5

[B82] Villalba J. J. Provenza F. (2009). Learning and dietary choice in herbivores. 62 399–406. 10.2111/08-076.1

[B83] Villalba J. J. Provenza F. Shaw R. (2005). Sheep self-medicate when challenged with illness-inducing foods. 71 1131–1139. 10.1016/j.anbehav.2005.09.012

[B84] Whitmore T. W. Turner B. L. (1992). Landscapes of cultivation in Mesoamerica on the eve of the conquest. 82 402–425. 10.1111/j.1467-8306.1992.tb01967.x

[B85] Willerslev R. Vitebsky P. Alekseyev A. (2015). Sacrifice as the ideal hunt: a cosmological explanation for the origin of reindeer domestication. 21 1–23. 10.1111/1467-9655.12142

[B86] Wyndham F. S. (2009). Spheres of relations, lines of interaction: subtle ecologies of the Rarámuri Landscape in Northern Mexico. 29 271–295. 10.2993/0278-0771-29.2.271

[B87] Yacobaccio H. D. Vilá B. L. (2016). A model for llama (*Lama glama* Linnaeus, 1758) domestication in the southern Andes. 51 5–13. 10.5252/az2016n1a1

[B88] Zeder M. A. (2011). The origins of agriculture in the Near East. 52(Suppl. 4) 221–235. 10.1086/659307

[B89] Zeder M. A. (2012). The domestication of animals. 2 161–190. 10.3998/jar.0521004.0068.201

[B90] Zeder M. A. (2015). Core questions in domestication research. 112 3191–3198. 10.1073/pnas.1501711112 25713127 PMC4371924

[B91] Zeder M. A. (2016). Domestication as a model system for niche construction theory. 30 325–348. 10.1007/s10682-015-9801-8 28839915

[B92] Zeder M. A. Smith B. D. (2009). A conversation on agricultural origins: talking past each other in a crowded room. 50 681–691. 10.1086/605553

[B93] Zohary D. Hopf M. Weiss E. (2012). Oxford: Oxford University Press 10.1093/acprof:osobl/9780199549061.001.0001

